#  “Everything for Her!”

**DOI:** 10.3325/cmj.2011.52.89

**Published:** 2011-02

**Authors:** Vesna Andrijević Matovac

I am in a hospital because of constrictive pericarditis that developed after pleural irradiation. I do not have enough physical strength to continue the story of my disease so I will tell you the story of our Association – an alliance of women with breast cancer and their friends and families that got together to help other women with breast and other types of cancer live with the disease and its therapy.

The Association of Women with Breast Cancer, Their Families and Friends “Everything for Her!” is a voluntary, non-government, and non-profit charity organization that provides information and psychological and logistical assistance to breast cancer patients and their families. My friends and I founded it on March 21, 2008. In time, our Association has became the model that integrates the ways society cares for the needs of individuals and their families, together with the motivation of professionals and volunteers to become involved in the overall advancement of mental health and to offer psychological and psycho-social support to patients.

Thanks to the Government of the Republic of Croatia and the City of Zagreb, the Association was given office space in Zagreb. With the help of everyone who participated in a series of charity events, the space was renovated, equipped, and presented to the public on May 21, 2010 at the official opening of the Center for Psychological Assistance to Breast Cancer Patients. The opening ceremony was attended by the Croatian President Ivo Josipović and Deputy Prime Minister Đurđa Adlešić on behalf of Prime Minister Jadranka Kosor (Figure 1).

**Figure 1:**
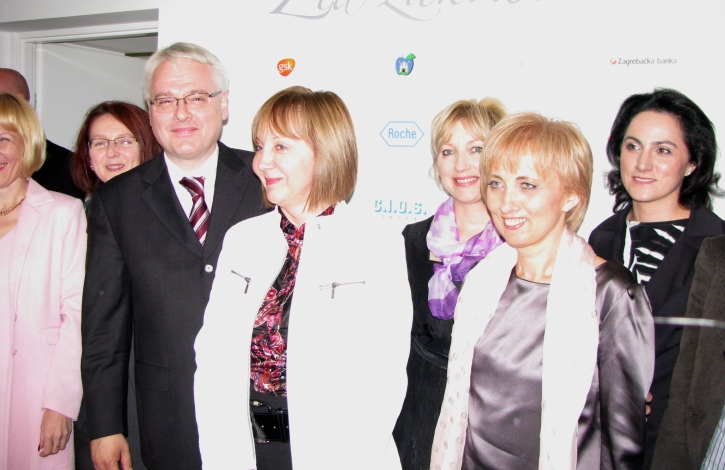
Opening of the Center for Psychological Assistance to Breast Cancer Patients in May 2010.

One important segment of the Association “Everything for Her!” are the activities aimed at preventing the illness and educating citizens and professionals. With great pride, we announce that we were granted funds from the European Commission for a project in 2011. The project is being carried out in association with the Croatian Nursing Council and aims at education, prevention/treatment, and research. The educational activities consist of working directly with medical staff, improving their skills and capacities for work with cancer patients, as well as training school psychologists to help the children of breast cancer patients. Preventative activities primarily involve the distribution of specialized information leaflets in hospitals and primary health care Centers in Croatia, whereas research includes a host of psycho-demographic tests to explore coping mechanisms of cancer patients.

## Psychological Assistance Center “Everything for Her!”

The standard treatment of cancer patients in Croatia does not include specific psychological help and support, even though the value and contribution of psychiatric and psychological support is undeniable. In developed countries, it is quite normal for psychologists and psychiatrists – experts in psycho-oncology – to be a part of the treatment team for cancer patients.

Our Psychological Assistance Center collaborates with the Zagreb University Hospital Center, especially with the Department of Medical Psychology, the Croatian Chamber of Psychologists, and the Croatian Nursing Council, with which we have also signed collaboration agreements. The Association and the Center also collaborate with prominent experts in the field of prevention, treatment, and palliative care. Even though the Association and Center primarily act in the City of Zagreb County, the activities will be spread to other areas around the country, because cancer knows no boundaries. Highly motivated psychologists with special backgrounds in psycho-oncology act as counselors, while the Center provides them with supervision services.

**Figure 2:**
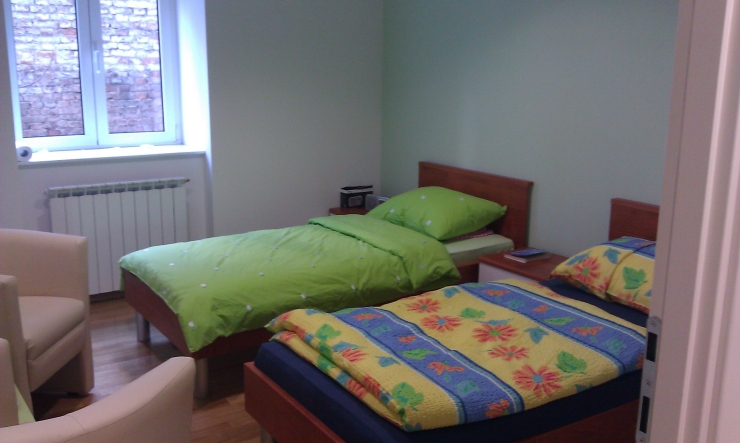
Accommodation for women coming for diagnosis and treatment to Zagreb hospitals.

Cancer patients are often exhausted, both emotionally and financially, and cannot afford to wait two months for psychiatric counseling or private treatment. The Center offers immediate help, as well as free accommodation for out-of-town patients during their stay in Zagreb for diagnosis or therapy (Figure 2).

We are pleased to see that social responsibility in Croatia for those in need is maturing, just as we were happy to see that our Psychological Assistance Center was officially recognized as a social organization, a place where society can offer a helping hand to individuals.

Women require psychological assistance throughout the entire treatment to make it efficient and as short as possible. Therefore, to enhance rehabilitation and the quality of life of women at all stages of the illness, we offer different programs on the removal and alleviation of difficulties and achieving psychological balance, adaptation, and integration of the illness into everyday life, psycho-education, individual counseling, group counseling, support groups, support to family and partners, legal counseling, psycho-sexual counseling, relaxation techniques, informational and educational activities, and nutrition counseling.

In only six months, the Center has had over 100 clients whose messages and personal progress during their treatment at the Center have given us the greatest possible motivation to keep going.

You can find all information on our activities at *http://www.svezanju.hr/* (in Croatian), or you can write to us at *info@svezanju.hr*. Our doors are open to all needing help and all with a good will to help.

